# Municipal sludge disposal emerges as a major, hidden, and rapidly growing atmospheric mercury source

**DOI:** 10.1073/pnas.2616833123

**Published:** 2026-11-04

**Authors:** Xingrui Cai, Mengqi Yang, Maodian Liu, Shijun Ma, Xinran Qiu, Qianru Zhang, Xuejun Wang

**Affiliations:** ^a^ Ministry of Education Laboratory of Earth Surface Processes, College of Urban and Environmental Sciences, Peking University, Beijing 100871, China; ^b^ The Bartlett School of Sustainable Construction, University College London, London WC1E 7HB, United Kingdom; ^c^ General Institute of Water Resources and Hydropower Planning and Design, Ministry of Water Resource, Beijing 100120, China; ^d^ School of Environment, Tsinghua University, Beijing 100084, China; ^e^ Institute of Carbon Neutrality, Peking University, Beijing 100871, China; ^f^ Nicholas School of the Environment, Duke University, Durham, NC 27708

**Keywords:** wastewater system, municipal sludge, atmospheric pollution, sustainability transitions, mercury

## Abstract

Wastewater treatment greatly reduces mercury pollution in aquatic ecosystems but does not eliminate it. Here, we identify municipal sludge disposal as a major, long-overlooked atmospheric mercury source. In China, sludge disposal emitted $42_{-10}^{+18}$ Mg in 2024, exceeding 10% of national anthropogenic emissions and ranking third after cement production and nonferrous metal smelting. These emissions have increased by nearly a thousandfold since the 1970s and are concentrated in densely populated urban areas, driven by the rapid expansion of sludge incineration, codisposal, and composting. Our findings reveal a cross-media pollution transfer embedded in sustainability transitions and call for sludge disposal to be incorporated into UNEP mercury budget and Minamata Convention assessments.

Mercury (Hg) is a neurotoxic pollutant of global concern (1). It undergoes long-range atmospheric transport, transforms into methylmercury in aquatic systems, and bioaccumulates in seafood. Anthropogenic activities have increased atmospheric Hg concentrations to three to five times preindustrial levels (2). Exposure through Hg-contaminated seafood causes ~30,000 premature deaths annually and economic losses of US$120 billion worldwide (3). These impacts underscore the urgent need for coordinated global Hg mitigation and effective implementation of the Minamata Convention (4).

China is the world’s largest anthropogenic source of Hg, contributing approximately 1/4 of global atmospheric emissions and aquatic releases (excluding artisanal and small-scale gold mining) (1, 5, 6). Recently, China has strengthened compliance with the Convention through coordinated carbon–pollutant controls and the phase-out of Hg-containing products. Updated estimates show that China’s atmospheric Hg emissions have declined since the early 2010s, driven mainly by reductions in coal combustion and metal smelting (7). Concurrently, wastewater treatment advances have substantially mitigated Hg contamination in aquatic environments and seafood, thereby lowering human exposure to methylmercury (8).

However, municipal wastewater treatment transfers Hg into sludge, whose fate remains poorly understood. From 1978 to 2024, municipal sludge production in China increased 320-fold, reaching ~20 Mt y^−1^ (8). Early weak regulation led to widespread stockpiling and disposal in simple landfills. More recently, China has shifted sludge disposal toward energy and resource recovery (9). These practices, however, may remobilize the Hg stored in sludge and re-emit it into the atmosphere. Such re-emissions remain absent from international and national Hg budgets, including the United Nations Environment Programme (UNEP) Global Mercury Assessment, and may offset gains from current Hg mitigation efforts (1).

In this Article, we quantify the spatiotemporal evolution of atmospheric Hg emissions associated with shifts in municipal sludge disposal in China over the past half-century. We compile a facility-level database integrating detailed information from 6,132 municipal wastewater treatment plants and 2,517 sludge disposal facilities. The database resolves sludge pretreatment and disposal technologies across wastewater treatment plants, incinerators, composting facilities, and codisposal kilns, enabling facility-scale estimates of Hg emissions across China.

## Results and Discussion

**Fig. 1.**
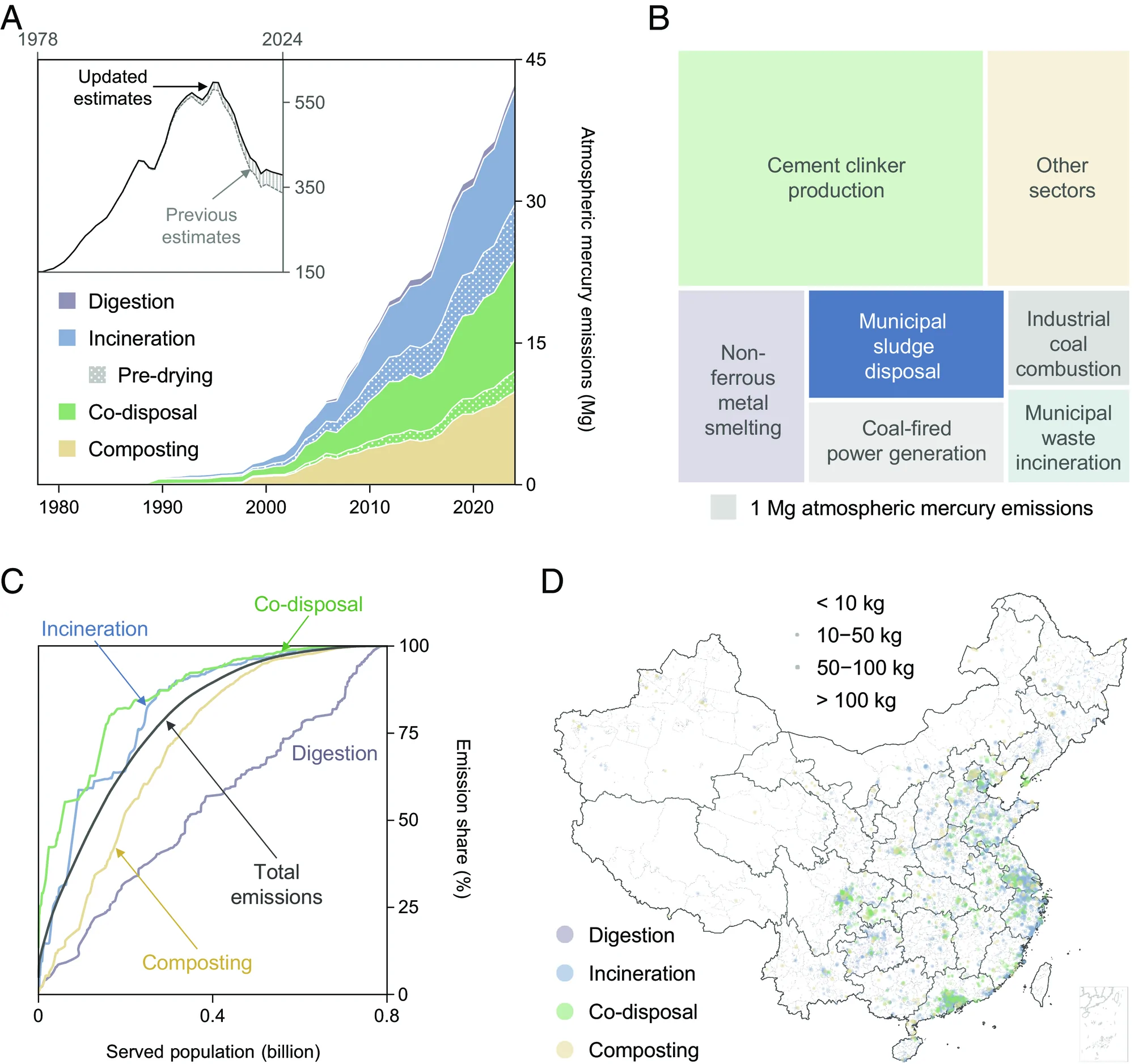
Rapid growth of atmospheric mercury emissions from municipal sludge disposal in China, 1978 to 2024. (*A*) Atmospheric Hg emissions from different disposal pathways. Predrying emissions are components of incineration and codisposal. Postapplication soil emissions are excluded from the sludge-disposal inventory. The inset compares existing estimates of China’s atmospheric Hg emissions with updated values including sludge disposal; data for other sectors are derived from literature (13). (*B*) Updated sectoral emissions in China in 2024. (*C*) Population-weighted Lorenz curve of sludge-disposal emissions in 2024, illustrating spatial–population inequality; cities are ranked by per-capita emission intensity. (*D*) Spatial distribution of atmospheric Hg emissions from sludge disposal facilities in 2024.

We reveal municipal sludge disposal as a critical yet overlooked source of atmospheric Hg. The Hg content of municipal sludge (mean: 3.5 μg g^−1^; minimum–maximum: 0.12 to 14 μg g^−1^) is 6 to 16 times higher than Hg-containing fuels such as coal (mean: 0.22 μg g^−1^) and municipal solid waste (mean: 0.60 μg g^−1^) (8, 10, 11). Accordingly, we estimate a national sludge Hg stock of 146 (133 to 167, 90% CI) Mg in 2024, 15 to 20 times the amount discharged in municipal wastewater effluent (12). Its disposal directly emitted 42 (32 to 60) Mg of Hg to the atmosphere in 2024, exceeding 10% of China’s anthropogenic emissions (Fig. 1*A*). These emissions surpass those from coal-fired power plants (32 Mg), ranking China’s third-largest source after cement production (141 Mg) and nonferrous metal smelting (49 Mg; Fig. 1*B*) (13).

In 2024, cities containing 40% of the population served by municipal wastewater systems (0.35 billion) accounted for 83% of sludge-related atmospheric Hg emissions (Fig. 1*C*). Large, densely populated, and economically developed cities tend to favor land-saving yet cost-intensive disposal options, such as incineration and codisposal, over landfilling (9, 14). Unlike in situ wastewater treatment, these thermal disposal facilities scale up to reduce marginal costs, resulting in cross-regional transfer of sludge and associated Hg emissions toward central cities (Fig. 1*D*). Consequently, this overlooked source may disproportionately impact residents in urban hubs, where contamination of soils and waters could intensify and accelerate Hg bioaccumulation and biomagnification through food webs.

**Fig. 2.**
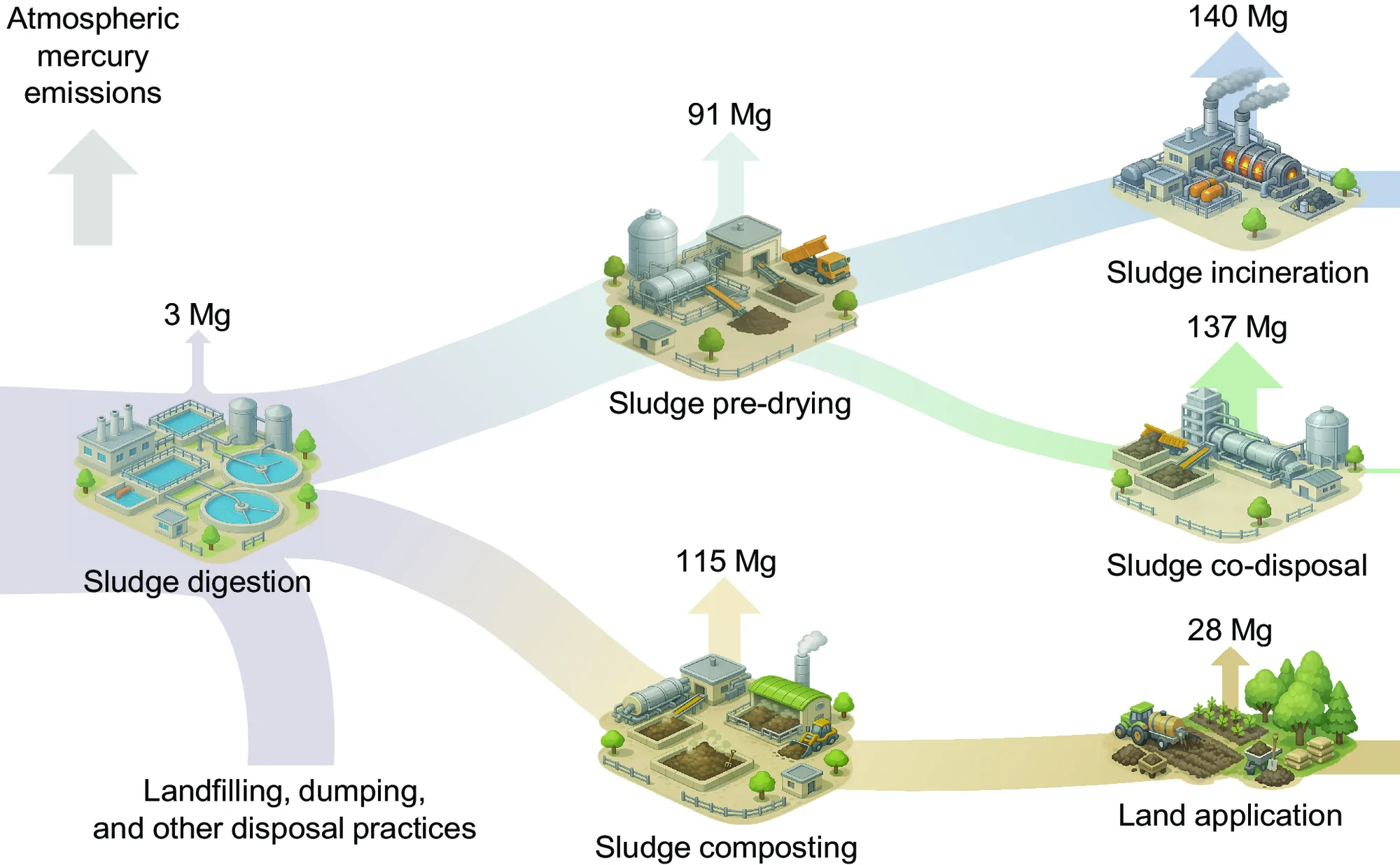
Total atmospheric Hg emissions from municipal sludge disposal in China, 1978 to 2024. Postapplication soil emissions are excluded from the sludge-disposal inventory.

Atmospheric Hg emissions from sludge disposal have increased rapidly, growing at an average of 29% per year since 2004. Owing to this omission, previous Chinese inventories likely underestimated cumulative atmospheric Hg emissions by 486 Mg (368 to 851 Mg) between 1978 and 2024 (Fig. 2) (11). Accounting for this source reveals a modest rebound in China’s anthropogenic atmospheric Hg emissions following their low point around 2020 (Fig. 1*A*) (13). Together, these trends identify the expanding municipal sludge-disposal practices as an emerging and critical target for atmospheric Hg mitigation.

Among municipal sludge disposal practices in China, incineration is the dominant source of atmospheric Hg emissions (18 Mg). Over the past 47 years, emissions from this pathway increased >800-fold, releasing a cumulative 206 Mg of Hg and accounting for 42% of all sludge-related atmospheric emissions (Fig. 2). In China, municipal solid-waste incineration is tightly regulated and typically equipped with advanced air pollution control devices (APCDs), achieving high Hg removal efficiencies (11). Sludge incineration, by contrast, operates under high-moisture and reducing conditions. These conditions destabilize halogen speciation, limit elemental Hg oxidation, and weaken the effectiveness of conventional APCDs. Moreover, sludge incineration generally requires predrying, which emitted 5.8 Mg of Hg in 2024, accounting for approximately 18% of thermal-treatment emissions and one-third of incineration-related emissions (Fig. 1*A*). Given the widespread absence of targeted APCDs, measures are urgently needed, including routing drying exhaust into the main incinerator or installing additional scrubbing systems.

Atmospheric Hg emissions from sludge codisposal (including its drying stage) increased sharply from <1 Mg in 2000 to 14 Mg in 2024. Recently, China has rapidly expanded the codisposal of municipal sludge in cement kilns for resource recovery. Mass-balance analyses indicated that recycling Hg-enriched fly ash into precalciner kilns further weakens Hg removal by APCDs (10); this suggests that emissions from these facilities are likely to continue increasing.

Sludge composting under warm, aerobic conditions promotes Hg demethylation, reduction, and volatilization, thereby reducing Hg toxicity in compost products and facilitating land application. Composting has therefore been widely promoted in China. Using flux-chamber measurements (Dataset S1), we estimate that sludge composting emitted 10 Mg of atmospheric Hg nationwide in 2024. Following compost application, soil Hg evasion increases sharply over the short term (~2 weeks). Peak fluxes (25 ng m^−2^ h^−1^) exceed the global mean terrestrial Hg flux (~0.1 ng m^−2^ h^−1^) by several hundred-fold (15). We estimate that this process contributed an additional ~2.4 Mg of atmospheric Hg in 2024 (Fig. 2). However, because postapplication evasion is classified as human-induced managed-soil emissions rather than direct sludge-disposal emissions, it is excluded from the sludge-disposal inventory (Fig. 1).

Overall, we identify municipal sludge disposal in China as a rapidly expanding yet long-overlooked source of atmospheric Hg, particularly as circular-economy policies advance. China’s municipal solid-waste incineration sector has expanded dramatically, and incineration capacity already exceeds waste generation in several economically developed regions, including the Yangtze and Pearl River Deltas (9). Surplus capacity may incentivize greater coincineration of Hg-rich sludge, further amplifying atmospheric Hg emissions in urban hubs. More importantly, ongoing landfill mining in China could remobilize Hg stored in legacy sludge deposits, triggering substantial short-term releases that elevate local exposure risks and perturb global Hg budgets (14). These findings underscore the need for pathway-specific controls, including Hg-specific abatement at incinerators and codisposal facilities and amendments during composting, such as biochar to immobilize Hg and lime to reduce its bioavailability.

Municipal wastewater treatment and sludge disposal remain essential, as poorly managed, pathogen- and nutrient-rich sludge poses serious public health risks and resource losses. Our findings show that advancing the Sustainable Development Goals (SDGs), particularly SDG 6 (Clean Water and Sanitation) and SDG 11 (Sustainable Cities and Communities), requires life-cycle improvements in environmental quality (SDG 3, Good Health and Well-being) while preventing cross-sectoral pollution transfer (SDG 10, Reduced Inequalities), thereby enabling cobenefits across sustainability goals.

Broadly, although the UNEP has incorporated aquatic Hg releases from municipal wastewater into global assessments, atmospheric Hg emissions from sludge disposal remain unquantified (1). As global waste and wastewater infrastructure expands, especially in rapidly urbanizing countries, this omission is likely to further bias global Hg budgets and threaten human well-being through increased dietary Hg exposure. Therefore, as conventional Hg sources are progressively controlled, sludge disposal and other nontraditional sources should be incorporated into global inventories and Minamata Convention compliance frameworks. We recommend: technically, revising Hg inventory toolkits to explicitly include sludge disposal as an emission source; policy-relevant, prioritizing this source in the Convention’s next global Hg assessment. Our study provides a methodological framework and emission parameters for quantifying these emissions in rapidly urbanizing regions worldwide. Such efforts would promote coherent cross-sectoral environmental governance and support a long-term balance among economic development, environmental quality, and public health.

## Materials and Methods

This study establishes a closed and internally consistent framework integrating data constraints, process-based modeling, spatiotemporal allocation, and uncertainty quantification. We compile detailed facility-level data for 6,132 municipal wastewater treatment plants, 2,150 incinerators, 220 composting facilities, and 147 cement kilns for sludge codisposal across China. Extensive experimental evidence on sludge Hg concentrations and release rates is integrated with machine-learning algorithms to quantify Hg flows. Detailed methods are provided in *SI Appendix*.

## Supplementary Material

Appendix 01 (PDF)

Dataset S01 (XLSX)

Dataset S02 (XLSX)

Dataset S03 (XLSX)

## Data Availability

All study data are included in the article and/or supporting information.
