# Supplementary material for: Municipal sludge disposal emerges as a major, hidden, and rapidly growing atmospheric mercury source
Source: Proc Natl Acad Sci U S A. 2026 Nov 4;123(45):e2616833123. doi: 10.1073/pnas.2616833123 (PMC13751679; doi:10.1073/pnas.2616833123)
Supplement: Supplementary file 1 [file pnas.2616833123.sapp.pdf]

PNAS

Supporting Information for

Municipal sludge disposal emerges as a major, hidden, and rapidly growing atmospheric mercury source

Xingrui Cai[1,2], Mengqi Yang[3,4], Maodian Liu[1,5,*], Shijun Ma[2], Xinran Qiu[1], Qianru Zhang[6], Xuejun Wang[1,5,*]

[1] Ministry of Education Laboratory of Earth Surface Processes, College of Urban and Environmental Sciences, Peking University, Beijing 100871, China

[2] The Bartlett School of Sustainable Construction, University College London, London WC1E 7HB, UK

[3] General Institute of Water Resources and Hydropower Planning and Design, Ministry of Water Resource, Beijing 100120, China

[4] School of Environment, Tsinghua University, Beijing 100084, China

[5] Institute of Carbon Neutrality, Peking University, Beijing 100871, China

[6] Nicholas School of the Environment, Duke University, Durham, NC 27708, USA

* Maodian Liu, Xuejun Wang

Email: maodian.liu@pku.edu.cn; xjwang@urban.pku.edu.cn

This PDF file includes:

Supporting text
Legends for Datasets S1 to S3
SI References

Other supporting materials for this manuscript include the following:

Datasets S1 to S3

Supporting Information Text
Extended Methods

This study employs a bottom-up framework that establishes a fully closed and internally consistent chain integrating data constraints, process-based modelling, spatiotemporal allocation, and rigorous uncertainty quantification. We compile data for 6,132 municipal wastewater treatment plants, including their locations, operation status, treatment technologies, capacities, sludge production, and sludge thickening, digestion, dewatering, and disposal practices. We used official statistics to ensure consistency with the underlying data used in the Global Mercury Assessment and existing China's Hg emission inventories(1–5), thereby ensuring direct comparison of the resulting estimates. Building on published work(6, 7), we evaluated multiple machine-learning algorithms. We selected the best-performing random forest model, trained on 714 plant-level sludge Hg measurements, to update estimates of sludge Hg concentrations and stocks in wastewater treatment plants during 1978–2024 (Dataset S2). The model achieved R^2 and mean absolute percentage error values of 0.86 and 0.28 on the training set, respectively, and 0.83 and 0.60 on the test set, respectively(6).

To characterize emissions from sludge disposal, we compile a new facility-level database covering 2,150 incinerators, 220 composting facilities, and 147 cement kilns for co-disposal. For each facility, the database records its location, commissioning year, capacity, and core technologies (Dataset S3). We geospatially match sludge disposal destinations of treatment plants to this updated database(8). Sludge is assigned annually to the nearest operating facility of the corresponding disposal practice, subject to capacity constraints and an empirical transport distance threshold (<100 kilometers), reflecting the limited economic feasibility of long-distance transport of wet sludge, consistent with reported operational practices(9, 10). Plants without any feasible match are treated as in situ disposal, consistent with historical on-site handling practices.

Hg release rates for each disposal practice are derived from experimental evidence in China (Dataset S1). The Hg release rate for co-disposal is derived from previous Hg mass-balance analyses covering thousands of kilns across China(11). For clarity, emissions from sludge incineration and co-disposal include only Hg originating from sludge, excluding co-fired coal, waste, limestone, or other materials (Dataset S2)(12–14). Under China's updated national standard in 2020, sludge-derived compost shall be used for landscaping and greening; the resulting atmospheric Hg emissions should therefore be classified as human-induced emissions from managed soils(15–17), and assigned to "Agriculture, Forestry and Other Land Use" following the 2019 Guidelines of Intergovernmental Panel on Climate Change (IPCC)(18). Given that our study focuses on Hg emissions from sludge disposal processes within the waste sector—corresponding to "Waste" in the Guidelines(19)—we discuss these post-application soil Hg emissions separately and exclude them from the total emissions to avoid inappropriate sectoral allocation. Atmospheric Hg emissions from (un)regulated landfilling are estimated at 0.4 Mg in

2024; given their minor contribution and large uncertainties associated with poorly constrained mechanisms, these emissions are excluded in the aggregated inventories.

This study compiles all available observations from the existing literature as possible to ensure methodological consistency and comparability across estimates (Dataset S1). As an external validation, we compared these rates with published measurements from Europe and North America for incineration(20–24), composting(25–27), and co-disposal(28–30). Their broad agreement provides an independent check on the representativeness of the adopted parameters, although facility-specific variability remains. As with most existing emission inventories(1, 2, 5, 31), however, the Hg release rates of various disposal pathways could not be independently validated against facility-level monitoring data. Extensive field measurements across disposal facilities, combined with detailed activity data, would help further constrain emission parameters and improve the accuracy and credibility of the inventory. Due to a lack of facility-level details, we did not further investigate the effects of technical and environmental variables, such as composting temperature and aeration.

For uncertainty analysis, we conduct 10,000 Monte Carlo simulations and report 90% confidence intervals (P5–P95). Following common practice(32, 33), official statistics (e.g., sludge production) are modeled as normally distributed with a coefficient of variation (CV) of 5%. Sludge Hg concentrations are modeled as log-normally distributed to account for their inherent skewness, with parameters derived from provincial datasets(6, 34, 35). In the simulations, we use nonparametric bootstrap resampling to propagate uncertainty in the Hg release rates of different disposal pathways, thereby avoiding potential biases introduced by fitting parametric distributions to small samples(36, 37). The observations used for resampling are provided in Dataset S1. Consistent with previous studies, we applied a 10% misassignment rate for linking wastewater treatment plants to disposal facilities; affected sludge is reassigned to the second-nearest feasible facility(8). To account for the greater uncertainty in long-term reconstruction, we followed earlier approaches and IPCC guidance by applying additional CV multipliers. Specifically, CVs are doubled for years before 2000 and increased fivefold for years before 1990, to avoid systematic underestimation of uncertainty in early years(32).

Dataset S1 (separate file). Atmospheric mercury emission characteristics of different municipal sludge disposal pathways.

Note: Release rates are defined as the ratio of atmospheric Hg emissions to the total Hg input from municipal sludge at a given disposal facility or site. Data sources are provided in the table.

Dataset S2 (separate file). Data on atmospheric mercury emissions associated with municipal sludge disposal in China.

Note: Atmospheric mercury emissions from sludge drying prior to incineration or co-disposal are a component of emissions from sludge incineration or co-disposal, respectively; these emissions should not be used to calculate the total emission due to double counting.

Dataset S3 (separate file). Basic data on municipal sludge disposal facilities in China.

Note: Basic data are sourced from the Public Platform for Automatic Monitoring Data of Municipal Solid Waste Incinerators (for incinerators); the National Pollutant Discharge Permit Management Information Platform (for composting facilities); and the China Cement Network's Cement Big-Data Platform (for co-disposal facilities).

SI References

1. UN Environment, “Global Mercury Assessment 2018” (UN Environment Programme, Chemicals and Health Branch, 2019).
2. X. Qiu, *et al.*, Declines in anthropogenic mercury emissions in the Global North and China offset by the Global South. *Nat. Commun.* **16**, 1179 (2025).
3. Y. Zhang, *et al.*, Improved Anthropogenic Mercury Emission Inventories for China from 1980 to 2020: Toward More Accurate Effectiveness Evaluation for the Minamata Convention. *Environ. Sci. Technol.* **57**, 8660–8670 (2023).
4. K. Liu, *et al.*, Highly Resolved Inventory of Mercury Release to Water from Anthropogenic Sources in China. *Environ. Sci. Technol.* **55**, 13860–13868 (2021).
5. D. G. Streets, Q. Zhang, Y. Wu, Projections of Global Mercury Emissions in 2050. *Environ. Sci. Technol.* **43**, 2983–2988 (2009).
6. X. Cai, *et al.*, China’s municipal wastewater policies enhanced seafood safety and offset health risks from atmospheric mercury emissions in the past four decades. *Nat. Food* **6**, 182–195 (2025).
7. P. Xu, *et al.*, Fertilizer management for global ammonia emission reduction. *Nature* **626**, 792–798 (2024).
8. Y. Guo, *et al.*, Benefits of infrastructure symbiosis between coal power and wastewater treatment. *Nat. Sustain.* **5**, 1070–1079 (2022).
9. A. Wang, *et al.*, Global data–water symbiosis reduces AI infrastructure’s carbon and water footprint. *Environ. Sci. Ecotechnology* **31**, 100702 (2026).
10. M. Marufuzzaman, S. D. Ekşioğlu, R. Hernandez, Truck versus pipeline transportation cost analysis of wastewater sludge. *Transp. Res. Part Policy Pract.* **74**, 14–30 (2015).
11. X. Cai, *et al.*, Establishment of High-Resolution Atmospheric Mercury Emission Inventories for Chinese Cement Plants Based on the Mass Balance Method. *Environ. Sci. Technol.* **54**, 13399–13408 (2020).
12. J. Guo, *et al.*, Temporal and spatial analysis of anthropogenic mercury and CO2 emissions from municipal solid waste incineration in China: Implications for mercury and climate change mitigation. *Environ. Int.* **178**, 108068 (2023).
13. Y. Duan, C. Zhao, Y. Wang, C. Wu, Mercury Emission from Co-combustion of Coal and Sludge in a Circulating Fluidized-Bed Incinerator. *Energy Fuels* **24**, 220–224 (2010).
14. Y. Sun, G. Chen, B. Yan, Z. Cheng, W. Ma, Behaviour of mercury during Co-incineration of sewage sludge and municipal solid waste. *J. Clean. Prod.* **253**, 119969 (2020).
15. M. Crippa, *et al.*, EDGAR (Emissions Database for Global Atmospheric Research) Community GHG Database. European Commission, Joint Research Centre (JRC). Deposited 1 January 2025.

16. A. Carpi, S. E. Lindberg, E. M. Prestbo, N. S. Bloom, Methyl Mercury Contamination and Emission to the Atmosphere from Soil Amended with Municipal Sewage Sludge. *J. Environ. Qual.* **26**, 1650–1655 (1997).
17. A. Carpi, S. E. Lindberg, Sunlight-Mediated Emission of Elemental Mercury from Soil Amended with Municipal Sewage Sludge. *Environ. Sci. Technol.* **31**, 2085–2091 (1997).
18. IPCC, *2019 Refinement to the 2006 IPCC Guidelines for National Greenhouse Gas Inventories, Volume 4: Agriculture, Forestry and Other Land Use*, E. Calvo Buendia, *et al.*, Eds. (Intergovernmental Panel on Climate Change, 2019).
19. IPCC, *2019 Refinement to the 2006 IPCC Guidelines for National Greenhouse Gas Inventories, Volume 5: Waste*, E. Calvo Buendia, *et al.*, Eds. (Intergovernmental Panel on Climate Change, 2019).
20. U.S. Environmental Protection Agency, "Final Emissions Factors for AP-42 Chapter 2, Section 2 - Sewage Sludge Incineration" (2026).
21. S. J. Balogh, Y. H. Nollet, Mercury mass balance at a wastewater treatment plant employing sludge incineration with offgas mercury control. *Sci. Total Environ.* **389**, 125–131 (2008).
22. Y. Cheng, S. Oleszek, K. Shiota, K. Oshita, M. Takaoka, Comparison of sewage sludge mono-incinerators: Mass balance and distribution of heavy metals in step grate and fluidized bed incinerators. *Waste Manag.* **105**, 575–585 (2020).
23. D. Pudasainee, Y. C. Seo, J. H. Kim, H. N. Jang, Fate and behavior of selected heavy metals with mercury mass distribution in a fluidized bed sewage sludge incinerator. *J. Mater. Cycles Waste Manag.* **15**, 202–209 (2013).
24. M. Takaoka, K. Oshita, M. Okada, T. Watanabe, K. Tanida, Mercury behaviour in flue gas from sewage sludge incinerators and melting furnace. *Water Sci. Technol.* **2017**, 782–790 (2018).
25. M. J. Caraballo-Laza, *et al.*, Composting as a Sustainable Approach for Managing Mercury-Contaminated Aquatic Biomass. *Toxics* **13**, 553 (2025).
26. B. Janowska, Remission of Mercury Compounds from Sewage Sludge Disposal. *Inż. Ekol.* **2016**, 49–57 (2016).
27. B. Janowska, K. Szymański, R. Sidełko, I. Siebielska, B. Walendzik, Assessment of mobility and bioavailability of mercury compounds in sewage sludge and composts. *Environ. Res.* **156**, 394–403 (2017).
28. D. Dajnak, K. D. Clark, F. C. Lockwood, G. Reed, The prediction of mercury retention in ash from pulverised combustion of coal and sewage sludge. *Fuel* **82**, 1901–1909 (2003).
29. M. H. Lopes, P. Abelha, J. F. S. Oliveira, I. Cabrita, I. Gulyurtlu, Heavy Metals Behavior during Monocombustion and Co-Combustion of Sewage Sludge. *Environ. Eng. Sci.* **22**, 205–220 (2005).

30. I. Gulyurtlu, H. Lopes, P. Abelha, I. Cabrita, J. F. S. Oliveira, The study of partitioning of heavy metals during fluidized bed combustion of sewage sludge and coal. *J. Energy Resour. Technol. Trans. ASME* **128**, 104–110 (2006).
31. Y. Cui, *et al.*, Integrating point sources to map anthropogenic atmospheric mercury emissions in China, 1978–2021. *Earth Syst. Sci. Data* **17**, 3315–3328 (2024).
32. Intergovernmental Panel on Climate Change, "Volume 3: Uncertainties" in *2006 IPCC Guidelines for National Greenhouse Gas Inventories*, (Institute for Global Environmental Strategies, 2006).
33. Risk Assessment Forum, U.S. Environmental Protection Agency, "Guiding Principles for Monte Carlo Analysis" (1997).
34. K. Liu, *et al.*, A Highly Resolved Mercury Emission Inventory of Chinese Coal-Fired Power Plants. *Environ. Sci. Technol.* **52**, 2400–2408 (2018).
35. L. Zhang, *et al.*, Updated Emission Inventories for Speciated Atmospheric Mercury from Anthropogenic Sources in China. *Environ. Sci. Technol.* **49**, 3185–3194 (2015).
36. Intergovernmental Panel on Climate Change, "Volume 1: General Guidance and Reporting" in *2006 IPCC Guidelines for National Greenhouse Gas Inventories*, (Institute for Global Environmental Strategies, 2006).
37. "EMEP/EEA air pollutant emission inventory guidebook 2019: Technical guidance to prepare national emission inventories" (European Environment Agency, 2019).
